# The Effect of Human and Mouse Fibroblast Feeder Cells on Cardiac Differentiation of Human Pluripotent Stem Cells

**DOI:** 10.1155/2012/875059

**Published:** 2012-01-19

**Authors:** Mari Pekkanen-Mattila, Marisa Ojala, Erja Kerkelä, Kristiina Rajala, Heli Skottman, Katriina Aalto-Setälä

**Affiliations:** ^1^Institute of Biomedical Technology, University of Tampere, Biokatu 12, 33520 Tampere, Finland; ^2^BioMediTech Biokatu 12, 33520 Tampere, Finland; ^3^Heart Center, Tampere University Hospital, Teiskontie 35, 33520 Tampere, Finland

## Abstract

Mouse embryonic fibroblasts (MEFs) and human foreskin fibroblasts (hFFs) are commonly used as feeder cells to maintain the pluripotent state of stem cells. The aim of the present study was to evaluate the effect of MEF and hFF feeders on the cardiac differentiation. Two human embryonic and two induced pluripotent stem cell lines were cultured on MEF and hFF before cardiac differentiation. The expression of Brachyury T was higher in cell lines cultured on MEF, than if cultured on hFF, suggesting enhanced mesoderm formation. However, significant positive influence of MEF feeders on cardiac differentiation was only seen with one cell line. Further, the ability of hFF to maintain pluripotency of stem cells originally cultured on MEF was quite poor. In conclusion, the cells behaved differently whether cultured on hFF or MEF feeders. However, the influence of the feeder cells on differentiation was less than the difference observed between the cell lines.

## 1. Introduction

The first human embryonic stem cell (hESC) line was derived in 1998 [1]. Currently, pluripotent stem cells can also be obtained from human somatic cells by reprogramming the cells with a defined set of factors [2, 3]. hESCs and induced pluripotent stem (iPS) cells require complex culture systems comprising of specialized media and mouse or human feeder cell layers that support the undifferentiated state and pluripotency [1, 4, 5]. Several feeder cell-free culture systems using extracellular matrix (ECM) components instead of feeder cell layers have also been developed [6–9].

Feeder cells support the growth of pluripotent stem cells by producing growth factors and providing adhesion molecules and ECM components for cell attachment. hESCs were originally derived and cultured on mouse embryonic fibroblast (MEF) feeder cell layers [1]. To overcome the problem of xenocontamination, feeder cells have also been derived from several human cell types, such as human foreskin fibroblasts (hFFs) or adult Fallopian tube epithelial cells [4, 10–14]. However, the ability of different types of human feeder cells to support the undifferentiated growth of hESCs varies [11, 15]. Activin A and basic fibroblast growth factor (bFGF) are key factors in maintenance the pluripotent state of stem cells [15, 16]. Mouse feeder cells express more Activin A than human feeder cells, but they do not express bFGF like human feeder cells [15]. When compared to human feeder cells, MEFs support better the undifferentiated growth of some hESC lines, whereas more spontaneous differentiation and a lower proportion of SSEA3 positive cells can be observed with human feeder cells [15].

hESCs and iPS cells can be induced to differentiate into cardiomyocytes by multiple methods [17–24]. We have previously demonstrated that hESC lines differ in their cardiac differentiation potential [25], and all the cell lines used in our earlier study had a relatively poor cardiac differentiation efficiency. The H7 hESC line is widely used in stem cell research, and it has been reported to have relatively good cardiogenic potential in cardiac differentiation studies [20, 26]. The major difference in the H7 cell line and hESC lines derived in our institute is that we have used human feeder cells, while H7 has been derived and cultured MEFs. Therefore, we hypothesized that the feeder cell type affects cardiac differentiation efficiency. To evaluate the effect of feeder cells on cardiac differentiation, we adapted our hESC line, Regea 08/017, to MEFs and H7 to hFF feeder cells. In addition, we compared the differentiation of two human iPS cell lines UTA.00106 and UTA.00112 on both feeder types.

## 2. Materials and Methods

### 2.1. Cell Culture

The hESC lines Regea 08/017 [27] and H7 (WiCell Research Institute) and the human iPS cell lines UTA.00106 and UTA.00112 (derived at the Institute of Biomedical technology, IBT, University of Tampere) were used. The study was conducted in accordance with the Ethics Committee of Pirkanmaa Hospital District to derivate and culture hESC and iPS cell lines. The iPS cell lines were induced from hFF (American Type Culture Collection, Manassas, VA) by retroviral transfection of Oct4, Sox-2, Klf4, and c-Myc [28].

#### 2.1.1. Adaptation

H7, UTA.00106 and UTA.00112 cell lines were normally cultured on MEFs (Millipore) and adapted to hFF (American Type Culture Collection) feeder cells for at least 5 passages. Regea 08/017 cell line was cultured originally on hFF feeder cells as previously described [27] and adapted to MEF feeder cells for at least 5 passages. The cell lines and adaptation procedure are presented in Table 1.

The hESC culture medium (KSR) comprised of knockout DMEM (Invitrogen, Carlsbad, CA, USA) containing 20% knockout serum replacement (KO-SR; Invitrogen), 1% nonessential amino acids (Cambrex Inc., Walkersville, MD, USA), 2 mM GlutaMax (Invitrogen), 50 U/mL penicillin/streptomycin (Cambrex), 0.1 mM 2-mercaptoethanol (Invitrogen), and 8 ng/mL bFGF (R&D Systems, Minneapolis, MN, USA). The undifferentiated state of the colonies was confirmed daily by morphologic analysis and with periodic testing for the expression of the stem cell markers Nanog, Oct4, SSEA4, and TRA-1-60. In addition, stem cell lines were also analyzed for their karyotypes which were all normal for lines H7, Regea 08/017, and UTA.00112, whereas UTA.00106 had an inversion in chromosome 12 (46, XY inv(12)).

### 2.2. Differentiation of Cardiomyocytes

Cardiomyocyte differentiation was induced by coculturing hESC and iPS cells with END-2 cells. END-2 cells were cultured as described earlier [29]. The passages used in differentiation experiments are described in Table 1. To initiate differentiation, undifferentiated cell colonies were dissected mechanically. The whole MEF feeder cell layer was removed carefully with pipette tip from the stem cell cultures and the undifferentiated colonies were detached by scraping with a cell scraper. The hFF feeder cell layer could not be removed using this method and therefore the stem cell colonies were detached from the feeder cell layer using a scalpel. Pieces of cell colonies containing a few hundred cells were placed on top of plated END-2 cells (30 pieces/well) in KSR culture medium with 3 mg/mL ascorbic acid and without serum, KO-SR, or bFGF [19]. The medium was changed after 5, 8, and 12 days of culturing. After 16 days, 10% KO-SR was added to the medium.

#### 2.2.1. Estimation of the Differentiation Efficiency

The differentiation efficiency was calculated by counting the number of beating areas and by cytospin analysis at day 21. Beating areas were counted from at least 24 wells of 12-well plates. For cytospin analysis, the cells from three wells of a 12-well plate (wells A1, B1, and C1) were trypsinized (20 min at +37°C). The total number of cells was determined and 5 × 10^5^ cells resuspended in a total volume of 12 mL PBS. The cells were spun at 800 rpm for 5 min onto polysine slides (Thermo Scientific) by the cytospin system (Cyto-Tech, Sakura). Cells were fixed with 4% paraformaldehyde (Sigma-Aldrich) and stained by anti-troponin T (Abcam). The percentage of troponin T-positive cells versus the total cell number (40, 6-diamidino-2-phenylindole [DAPI] staining of nuclei) was determined by counting at least 1500 cells (3 × 500 cells). Counted fields were randomly selected in the DAPI channel and under by using 20x magnification.

### 2.3. Immunocytochemistry

#### 2.3.1. Characterization of Undifferentiated Colonies

hESC and iPS cell colonies were fixed in 4% paraformaldehyde and stained with primary antibodies Oct4 (1 : 400, R&D Systems), Nanog (1 : 200, R&D Systems), TRA-1-60 (1 : 200, Millipore), and SSEA4 (1 : 200, Santa Cruz Biotechnology). Alexa Fluor 568 and 488-conjugated donkey anti-goat and anti-mouse antibodies were used as secondary antibodies (1 : 800, Invitrogen). Cells were mounted with Vectashield (Vector Laboratories) containing DAPI for staining nuclei.

#### 2.3.2. Characterization of Beating Cells

Beating areas were dissociated from cocultures 21 days after plating as previously described [25]. Immunocytochemical staining was performed with primary antibodies anticardiac troponin T (1 : 500, Abcam), antiventricular *α*-myosin heavy chain (*α*MHC) (1 : 50, Chemicon), and anti-Connexin 43 (1 : 1500, Chemicon).

### 2.4. Assessment of the Germ Layer Markers by Quantitative RT-PCR

Quantitative RT-PCR was performed according to the standard protocols on Abi Prism 7300 instrument (Applied Biosystems, Foster City, CA, USA). All cells from two END-2 coculture wells were collected and pooled at 0, 3, 6, and 12 days after initiating coculture. Total RNA was isolated with a NucleoSpin RNA II kit including DNAse treatment (Machery-Nagel, Duren, Germany). The concentration and quality of RNA were monitored spectroscopically (Nanodrop, Wilmington, DE, USA) and 1 *μ*g of total RNA was transcribed to cDNA in a total volume of 20 *μ*L with High Capacity cDNA Reverse Transcription Kit (Applied Biosystems). The PCR reaction comprised of 0.3 *μ*L cDNA, 7.5 *μ*L of 2x PCR Mastermix (Applied Biosystems), and 300 nM of each primer. Primer sequences were described previously [25]. In addition, the expression of Oct4 (POU5F) was determined by using the same cDNA, but the detection was performed by using the primer-probe sets NM_053275.3 for RPLP0 (endogenous control) and Hs00999632_g1 for POU5F (Oct4; Applied Biosystems).

*C*_*τ*_ values were determined for every reaction and the relative quantification was calculated with the 2^−ΔΔ*C*_*τ*_^ method [30]. The data were normalized to the expression of the endogenous control (RPLP0). The 0-day MEF sample of each cell line was used as the calibrator for differentiation studies and H7 (MEF) 0 d p43 sample for undifferentiated state analysis. Two biological replicates from each cell line were analyzed in triplicate. Results are shown as the mean values of both biological replicates.

### 2.5. Statistical Analysis

Quantitative RT-PCR data is presented as mean values +/− standard deviation. Statistical significance for qPCR data was determined by using Mann-Whitney test, and *t*-test was used for differentiation efficiency data. Difference was considered to be statistically significant with the *P* value of <0.05.

## 3. Results

### 3.1. Culture Adaptation

hESC line H7 and human Ips cell lines UTA.00106 and UTA.00112 were initially derived and maintained on MEF feeder cells and adapted to grow on hFF feeder cells for at least five passages. On the contrary, Regea 08/017 was established and originally cultured on hFF feeder cells and, therefore, adapted onto MEF for at least five passages as presented in Table 1. Regea 08/017 cells are usually passaged manually. However, to establish more similar culture conditions for the cell lines in the present study, these cells were also passaged enzymatically on hFF feeder cells.

Regea 08/017 cells grew well on MEF feeder cells with enzymatic passaging and the morphology of the colonies resembled H7, UTA.00106 and UTA.00112. Adaptation from MEF to hFF feeder cells, however, was more problematic. Overall, cells grew well and formed proper colonies but based on the visual inspection, the cells were more differentiated on hFF than on MEF feeder cells (data not shown). Compared to manual passaging, enzymatic passaging gives rise to greater amount of cell colonies. The higher amount of colonies might alter the feeder cell layer to detach from the cell culture well. This phenomenon was observed with hFF feeder cells in the present study.

### 3.2. Assessment of the Ability of MEF and hFF to Maintain the Undifferentiated State of Stem Cells

**Figure 1:**
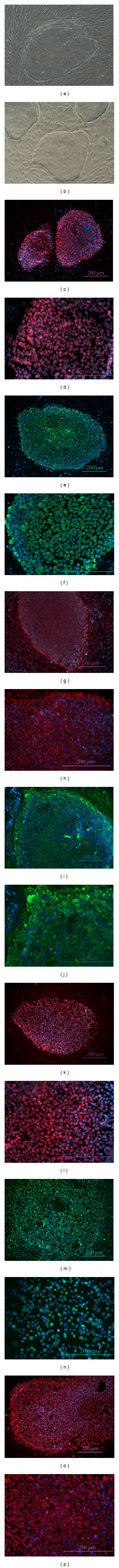
Undifferentiated H7 hESC colonies (on passage 15) 3 days after passaging on hFF feeder cells (a) and after 55 passages on MEF feeder cells (b). H7 cells passaged 11 times on hFF feeder cells expressed Nanog (c and d), Oct4 (e and f), SSEA4 (g and h), and TRA-1-60 (i and j). H7 cells on passage 45 on MEF feeder cells expressed Nanog (k and l), Oct4 (m and n), and SSEA4 (o and p). Scale bars 200 *μ*m.

**Figure 2:**
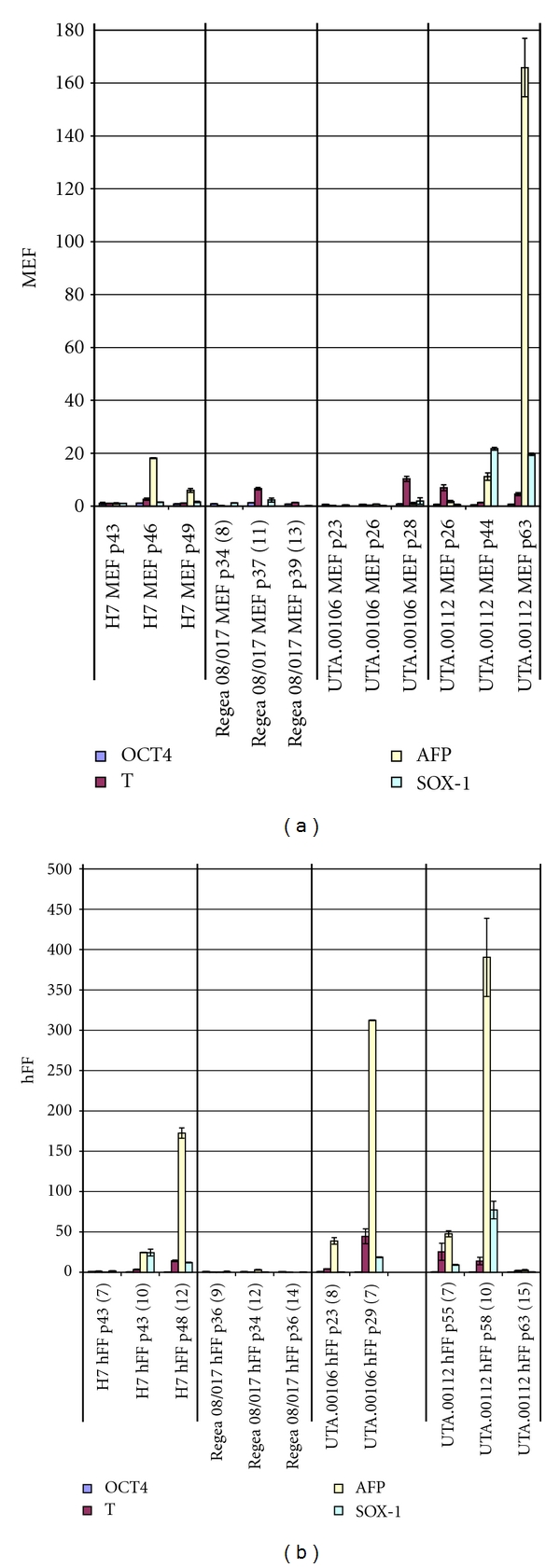
Relative gene expression levels of the undifferentiated stem cell lines on MEF (a) and on hFF (b). The H7 MEF p 43 sample has been used as the calibrator for all samples. Regea 08/017 has modest and steady expression levels of germ layer markers, whereas the expression levels in the iPS cell lines vary between passages.

Based on the immunocytochemistry of pluripotent markers, both feeder cell layers supported the hESC and iPS cells to maintain their pluripotent state (Figure 1). Performed qPCR studies also supported immunocytochemistry results. Oct4 expression was constant in all cell lines between passages and on both feeder cells. Basal expression levels of germ layer markers were also tested by qPCR. According to these results, Regea 08/017 had a low basal expression of Brachyury T, AFP, and Sox-1 on both of the feeder cell types and at all passages studied (Figure 2). However, increased germ layer marker expression was observed in the H7 cell line cultured on hFF compared to H7 cultured on MEFs (Figure 2). The expression of Brachyury T, AFP, and Sox-1 increased during culture of H7 on hFFs between passages p43 (7 passages on hFF feeders) and p48 (12 passages on hFF feeders) (*P* < 0.05). The expression of Brachyury T, AFP and SOX-1 was also significantly higher in H7 cells on hFF feeders than in H7 cultured on MEF feeders (*P* < 0.05).

The gene expression levels of germ layer markers in the iPS cell lines UTA.00106 and UTA.00112 were quite variable. Increase in germ layer marker expression levels was more obvious in hFF cultures but especially for UTA.00112 expression levels of Brachyury T, AFP, and Sox-1 fluctuated at the undifferentiated state on both of the feeder cell types (Figure 2).

### 3.3. Assessment of Germ Layer Markers during Cardiac Differentiation

**Figure 3:**
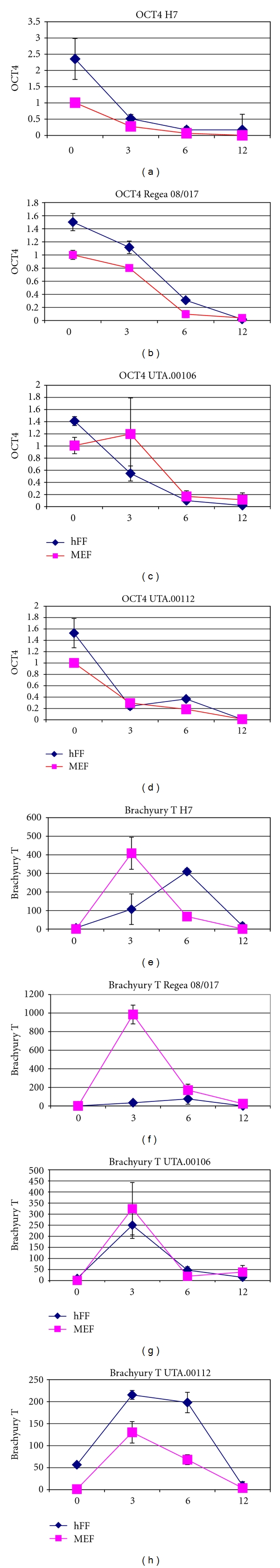
Oct4 (a–d) and Brachyury T (e–h) expression levels of H7, Regea 08/017, UTA.00106, and UTA.00112 cell lines on MEF and hFF feeder cells.

Oct4 expression decreased rapidly during differentiation (Figures 3(a)–3(d)), reaching a constant low level after day 6 in all the cell lines studied (*P* < 0.05). Oct4 expression decreased more slowly in Regea 08/017(hFF) compared to H7 (MEF) and H7 (hFF) and UTA.00106 (hFF) at day 3 (*P* < 0.05), and the differences were still significant on day 6 when compared to H7 (MEF) (*P* < 0.05).

Mesodermal marker Brachyury T expression (Figure 3(e)) was significantly higher at day 3 on H7 cultured on MEF than H7 cultured on hFF (*P* < 0.01). Similarly, Brachyury T expression was higher for Regea 08/017 cultured on MEFs than for Regea 08/017 cultured on hFFs (*P* < 0.01) (Figure 3(f)). Brachyury T expression peaked later (at day 6) in H7 (hFF) than in H7 (MEF). Regea 08/017 (hFF) had a similar expression curve having the expression peak at day 6. Expression levels for UTA.00106 were similar for both of the feeder cell types, peaking at day 3 (Figure 3(g)). However the curve for UTA.00112 was broader. In addition, for UTA.00112 on hFF culture, the level of Brachyury T expression was elevated when compared to MEF cultures, but the difference was already seen at day 0 (Figure 3(h)).

The expression curve for AFP was similarly shaped for all cell lines studied, having modest increase before day 6 but increasing strongly after day 6 (data not shown). Sox-1 expression trend was also similar in the cell lines, levels increasing from day 3 to 12 (data not shown).

**Figure 4:**
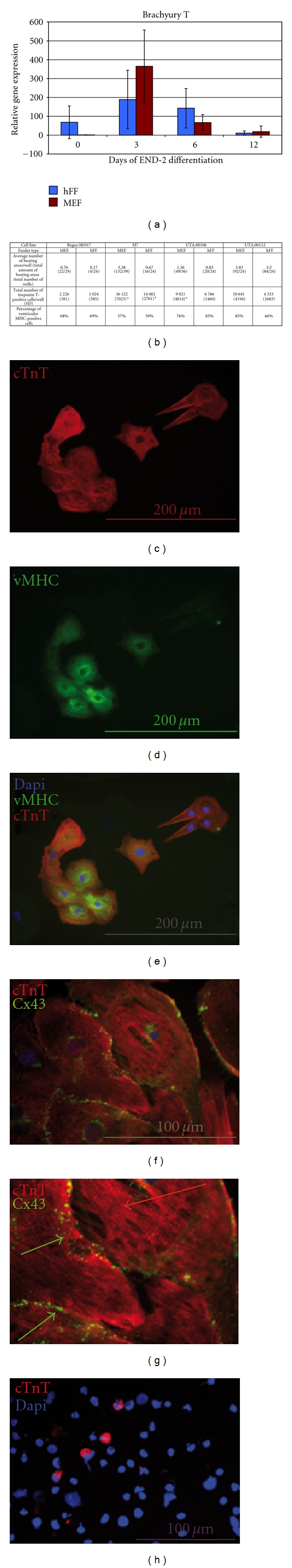
(a) Comparison of Brachyury T expression levels of MEF and hFF cultures. The differences of Brachyury T expression levels between hFF and MEF cultures on days 3 and 6 are statistically significant. In addition, the difference in expression levels of Brachyury T of MEF cultures between days 3 and 6 is statistically significant. Table in (b): Differentiation data of H7, Regea 08/017, UTA.00106, and UTA.00112 cell lines on MEF and hFF feeder cells. For H7, the difference between troponin T-positive cells is statistically significant, (*P* < 0.01). (c–h) Characterization of the differentiated cells. Troponin T (c), ventricular *α*-myosin heavy chain (d), and merged image with DAPI staining (e). Scale bar, 200 *μ*m. (f) Merged image with Troponin T (red) and Connexin 43 (green) revealing gap junctions between the cells. Scale bar 100 *μ*m. (g) Inset of image (f). (h) Cytospin slide of H7 (MEF) stained by troponin T (red) and DAPI (blue). Scale bar, 100 *μ*m.

Significant difference in Brachyury T expression level and the peak in expression was observed when the qPCR data from END-2 differentiation samples was analysed by combining the cells cultured on MEF and cells cultured on hFF (Figure 4(a)). In MEF cultures, the expression of Brachyury T at day 3 is higher than in hFF cultures (*P* < 0.05), and the expression level also decreased significantly more by day 6 (*P* < 0.05). Similar clear peaking at day 3 was not observed in hFF cultures, and no difference between days 3 and 6 was observed in hFF cultures. On the contrary, the level of Brachyury T was higher in hFF cultures at day 6 than in MEF cultures (*P* < 0.05).

### 3.4. Cardiac Differentiation Efficiency

The cardiac differentiation rate was estimated by counting the number of beating areas from at least 24 wells of 12-well plate. In addition, the percentage of troponin T-positive cells was quantified using the cytospin method. Both methods gave equivocal results, which are summarized in Figure 4(b). H7 and UTA.00112 cell lines had the best differentiation efficiency in this study. The iPS cell line UTA.00106 had also a relatively good, while Regea 08/017 had a poor, cardiac differentiation efficiency. Adaptation to the hFF feeder cells decreased the differentiation efficiency of H7 (from 3.38 to 0.67 beating areas per well) and UTA.00106 (from 1.36 to 0.83 beating areas per well) cell lines. A modest decrease in UTA.00112 was also observed (from 3.83 to 3.5). Respectively, adaptation to MEF feeder cells increased the differentiation rate of the Regea 08/017 line (from 0.17 to 0.76 beating areas per well).

As mentioned earlier, to support the differentiation efficiency determination, the number of troponin T-positive cells was determined, by cytospin analysis. Even though a similar amount of undifferentiated cells was plated on END-2 cells, after 21 days of coculturing, the total cell number in each well varied notably between the cell lines. The differences were also obvious by visual inspection. After 3 weeks of differentiation H7, UTA.00106, and UTA.00112 formed large cell structures, whereas the growth of Regea 08/017 was more restricted. This phenomenon was observed with both of the feeder cell types. In addition, cell growth was not affected by feeder cell type. The total number of cells in a well after 3 weeks in differentiation was the lowest in the Regea 08/017 line on both feeder cell types. When this data was combined with the cytospin analysis, the average number of cardiomyocytes in a well was calculated (Figure 4(b)). The amount of troponin T-positive cells was significantly higher for H7 (MEF) (36 122 cells) than H7 (hFF) (14 482 cells) (*P* < 0.01). The differentiation efficiency determined by the cytospin methods of iPS cell lines UTA.00106 and UTA.00112 did not differ between the MEF and hFF cultures. For the hESC line Regea 08/017, the number of cells in total and the number of troponin T-positive cells were notably lower when compared to H7. In addition, in Regea 08/017 the amount of troponin T-positive cells was slightly but not significantly higher when cultured on MEFs (2226) than on hFFs (1024) cultures (*P* = 0.087).

### 3.5. Immunocytochemistry

Beating cells from every cell line used in the study were positively stained by cardiac troponin T, and the staining revealed a striated pattern (Figures 4(c)–4(g)). In addition, Connexin 43 staining revealed gap junction structures between the cells (Figures 4(f)-4(g)). Most of the troponin T-positive cells were also positive for ventricular *α*-MHC (Figures 4(d)-4(e)) revealing the percentage of ventricular cells from the whole cardiomyocyte population (Figure 4(b)). The percentage of ventricular cardiomyocytes was independent of the feeder cell type. Regea 08/017, H7, UTA.00106, and UTA.00112 formed a mean of 68.5%, 58%, 79.5%, and 74.5% cardiomyocytes of ventricular type, respectively.

## 4. Discussion

It has been demonstrated earlier that hESC lines differ in their cardiac differentiation potential [25]. However, the cardiac differentiation efficiency for all the cell lines used in that study was relatively poor. On the contrary, the hESC line H7 is widely used in stem cell research and in cardiac differentiation studies with a relatively good cardiogenic potential [20, 26], and it also had the best differentiation efficiency in the present study. The major difference in the H7 cell line and hESC lines derived in our institute is that we have used human feeder cells while H7 has been initially derived and cultured on MEFs. Therefore, we hypothesized that the feeder cell type has an effect on cardiac differentiation. To evaluate the effect of feeder cells on cardiac differentiation more thoroughly, we adapted one of our hESC lines, Regea 08/017 to MEFs, and H7 cell line to hFF feeder cells. In addition, we compared the cardiac differentiation of two human iPS cell lines UTA.00106 and UTA.00112 on both feeder types.

Cardiomyocyte differentiation can be predicted by the transient expression of the early mesodermal marker Brachyury T. Brachyury T expression peak is normally detected at the time point of 3 days in END-2 cocultures [31]. Indeed, increased Brachyury T expression was seen when, for example, hESC line Regea 08/017 was cultured on MEF feeder cell layer compared to hFF feeders, indicating the elevated mesodermal differentiation. In addition, the number of beating areas for Regea 08/017 increased after MEF adaptation but the difference in the number of Troponin T positive cardiomyocytes did not reach statistical significance. However, the culturing of H7 on hFF feeder cells decreased the differentiation efficiency and also the number of troponin T-positive cells significantly. In addition, Brachyury T expression level was, respectively, decreased in H7 (hFF) compared to H7(MEF). However, the number of beating areas per well is a crude indication of differentiation efficiency. A beating area may be formed by a few to several thousands of cardiomyocytes [19]. Therefore, the cytospin method in combination with immunocytochemistry was used to support the differentiation efficiency data. Indeed, for example, the number of Troponin T-positive cells for the iPS cell line UTA.00112 was notably low if compared to the number of beating areas. This might be explained by the size of the beating areas and by the total number of cells in a well.

The differentiation efficiency of H7 cultured on MEFs was the highest in our study, resulting in 3.38 beating areas per well and on the average of 36 122 cardiomyocytes. In addition to H7 (MEF), the iPS cell lines UTA.00106 and UTA.00112 cultured on MEFs had a relatively good differentiation rate with 1.36 beating areas (9021 cardiomyocytes) and 3.83 beating areas (10 641 cardiomyocytes) per well, respectively. These numbers are similar to the previously reported results of END-2 cocultures of the HES-2 line, which formed a mean of 2.7 beating areas per well in serum-free medium [19].

In general, the cardiac differentiation of the human iPS cell lines UTA.00106 and UTA.00112 were similar to that of hESC lines; beating areas occurred on the same timescale and were morphologically alike. This is consistent with previous reports where iPS cells were differentiated to cardiomyocytes using the END-2 and embryoid body methods [22–24]. For iPS cell lines, the notable observation made in the present study was that feeder cell type did not have a significant effect on the cardiac differentiation efficiency.

One potential factor affecting cardiac differentiation efficiency might be Activin A. Activin A is essential for undifferentiated growth of hESC and is expressed more by MEFs than hFFs [15]. In addition, it affects the expression of pluripotency genes such as Oct4 and Nanog [16]. The enhanced Oct4 expression was not observed on hESC or iPS cells maintained on MEF compared to hFF. However, Activin A also induces the expression of growth factors such as Nodal, Wnt3, bFGF, and FGF-8 [16]. In addition, it has been used in combination with BMP-4, to induce cardiac differentiation with the formation of a primitive streak-like population and mesoderm [20, 21]. Based on the Brachyury T expression, mesoderm formation was more efficient in H7 and Regea 08/017 cell lines cultured on MEF than when cultured on hFF.

In addition, when the MEF and hFF cultures were compared, MEF-cultured cells had elevated Brachyury expression levels on day 3 and had a clear expression peak at this time point. As previously reported, Brachyury T expression peaks at day 3 and the delayed expression is indicative of poor cardiac differentiation [32]. Our results support this finding, as the expression peak was sharper and occurred earlier for MEF than hFF cultures, and the differentiation efficiency seemed to be higher in the MEF cultures.

 A notable difference between the cell lines studied was on the rates of the cell proliferation. At the initiation of coculturing, similar amounts of cells were plated on END-2 cells. However, after 21 days of coculturing, the total number of cells in Regea 08/017 cocultures was much lower than the number of cells in other cell lines thus resulting also smaller cardiomyocyte number. Activin A might also have a role in enhancing cell proliferation [33]. Therefore, the increased level of Activin A could explain better cell proliferation of H7 and iPS cell lines which have been originally derived and cultured on MEFs. In addition, the proliferative capability might also be cell line-specific, because adaptation from hFF to MEF did not have such an effect to total cell number. Regea 08/017 on MEF did not reach the proliferation level of UTA.00106, UTA.00112 or H7. A similar phenomenon was observed in our previous study, where differentiation on embryoid bodies was monitored; the growth of embryoid bodies varied between hESC lines [34].

Based on immunocytochemistry, both the MEF and hFF feeder cells maintained the undifferentiated growth of pluripotent stem cells. From the germ layer marker expression levels, however, it can be concluded that if the line has been derived and maintained on hFF, hFF feeder cells are able to maintain the pluripotent state and prevent differentiation. On the contrary, when H7, which has originally been cultured on MEFs, was grown on hFF feeder cells for 7 to 12 passages the expression of germ layer markers (Brachyury T, Sox-1, and AFP) increased. In addition, similar trend was seen with iPS cells. Therefore, the results indicate that hFF cannot maintain the undifferentiated state of H7 as effectively as MEF. This is consistent with earlier reports, where mouse feeder cells were more efficient for maintaining the undifferentiated state of hESC compared to human feeder cells [15]. Nevertheless, iPS cell lines tended to be more variable when compared to hESC lines according to qPCR results, and this was seen with both of the feeder cell types. This phenomena might be due to the genomic instability of iPS cells which has been discussed recently [35–37].

Differentiated beating cells were troponin T positive, and most of the beating cells were also ventricular *α*MHC-positive. The percentage of ventricular-type cardiomyocytes varied slightly between cell lines, UTA.00106 producing 79.5%, UTA.00112 74.5%, Regea 08/017 68.5%, H7 58%, and Regea 08/017 68.5% of the ventricular type of cells. A similar results were reported earlier, in a study where the hES2 line produced 64% ventricular cardiomyocytes and H1 produced 35%, [38]. In the previous report [38], the classification was based on action potential studies, whereas in the present report we used solely immunocytochemistry results. In our previous study, immunocytochemistry and action potential profiling provided congruent results [39].

## 5. Conclusions

The aim of the present study was to evaluate the effect of MEF and hFF feeder cells on cardiac differentiation. Four pluripotent stem cell lines were used: hESC lines Regea 08/017 and H7 and iPS-cell lines UTA.00106 and UTA.00112. The MEF feeders supported the cardiac differentiation better, but a significant effect on cardiac differentiation efficiency was seen with only one cell line used, namely H7. Brachyury T expression was elevated and a sharper expression peak at day 3 of differentiation was observed when cells were cultured on MEFs than if they were cultured on hFFs. This aforementioned sharp expression peak is an indicator of enhanced mesoderm formation and has been reported to be favourable to cardiac differentiation [32].

Even though both of the feeder cell types were able to support the undifferentiated state of the stem cells, increased expression levels of germ layer markers were observed especially in the hFF cultures, if the cells had been originally maintained on MEFs. Therefore, we conclude that the original feeder layer is important and the cells maintained on MEFs are not easily adapted on hFF feeders. The change from hFF to MEF was more successful. The germ layer marker expression in iPS cell lines was more variable compared to hESCs in undifferentiated state. However, iPS cells differentiated into functional cardiomyocytes similarly to hESCs.

## Figures and Tables

**Table 1:** Human pluripotent stem cell lines used in present study.

Stem cell line	Original feeder type	Adapted to	Code for the line	Passages used in differentiation
H7	MEF	—	H7 (MEF)	39–43 and 53
		hFF	H7 (hFF)	41–43 (cultured on hFF for 5–7 passages)
UTA.00106	MEF	—	UTA.00106 (MEF)	18–23
		hFF	UTA.00106 (hFF)	20–23 (cultured on hFF for 5–8 passages)
UTA.00112	MEF	—	UTA.00112 (MEF)	44-45 and 63
		hFF	UTA.00112 (hFF)	55–57 (cultured on hFF for 7–9 passages)
Regea 08/017	hFF	—	Regea 08/017 (hFF)	32–34
		MEF	Regea 08/017 (MEF)	31–36 (cultured on MEF for 5–9 passages)
